# The oblique perspective: philosophical diagnostics of contemporary life sciences research

**DOI:** 10.1186/s40504-017-0047-9

**Published:** 2017-03-04

**Authors:** Hub Zwart

**Affiliations:** Department of Philosophy and Science Studies (Chair), Faculty of Science, Institute for Science, Innovation and Society (ISIS), Radboud University Nijmegen, P.O. Box 9010, Nijmegen, GL 6500 The Netherlands

## Abstract

This paper indicates how continental philosophy may contribute to a diagnostics of contemporary life sciences research, as part of a “diagnostics of the present” (envisioned by continental thinkers, from Hegel up to Foucault). First, I describe (as a “practicing” philosopher) various options for an oblique (or symptomatic) reading of emerging scientific discourse, bent on uncovering the basic “philosophemes” of science (i.e. the guiding ideas, the basic conceptions of nature, life and technology at work in contemporary life sciences research practices). Subsequently, I outline a number of radical transformations occurring both at the object-pole and at the subject-pole of the current knowledge relationship, namely the technification of the object and the anonymisation or collectivisation of the subject, under the sway of automation, ICT and big machines. Finally, I further elaborate the specificity of the oblique perspective with the help of Lacan’s theorem of the four discourses. Philosophical reflections on contemporary life sciences concur neither with a Master’s discourse (which aims to strengthen the legitimacy and credibility of canonical sources), nor with university discourse (which aims to establish professional expertise), nor with what Lacan refers to as hysterical discourse (which aims to challenge representatives of the power establishment), but rather with the discourse of the analyst, listening with evenly-poised attention to the scientific files in order to bring to the fore the cupido sciendi (i.e. the will to know, but also to optimise and to control) which both inspires and disrupts contemporary life sciences discourse.

*“More detail is needed about the research’s methodology. It is not sufficiently clear how the main objectives of the research can be reached”* (anonymous reviewer of a grant proposal)

## Introduction

For continental philosophers working in interdisciplinary environments and committed to assessing the philosophical and societal dimensions of contemporary technoscience, the methodology section of grant proposals may pose a challenge. How to explain (to reviewers from the natural science, the social sciences or more mainstream areas of philosophical inquiry such as author studies or biomedical ethics) what continental philosophers studying technoscience actually do? Although standard phrases (“discourse analysis”, “desk research”, etc.) are available for such occasions, compared to the methodologies of the social sciences, or even bioethics, the tools and methods for this type of work seem much less clearly defined. To what extent and in what way may continental philosophical inquiry be considered “applicable” or even “replicable”, for instance?

Although the signifier “continental philosophy” began its career as a pejorative term and remains difficult to define, a family likeness is nonetheless discernible among adepts (Critchley 2001; Glendinning 2006; Zwart, Landeweerd and Lemmens 2016), including the conviction that outstanding techno-scientific developments (such as the Human Brain Project or the synthetic cell) may be regarded as symptoms or exemplifications of the zeitgeist, providing relevant input for a diagnostics of the present, as Hegel^1^ phrases it and perhaps even pointing to a “metaphysical mutation” (Houellebecq 1998), a new “dawn of day” (Nietzsche 1881/1980). Yet, compared to other areas of inquiry, continental philosophical reflections tend to stay relatively close to activities human beings already engage in in every-day, non-academic settings, such as talking, reading, listening and thinking. Taking notes, asking questions, paying attention, visiting laboratories and discussing the drawbacks and benefits of emerging technologies can hardly be considered an idiosyncrasy of academic philosophers, although they may claim to do this in a comparatively consistent, critical and thoughtful manner. When it comes to reading, for instance, continental philosophers have various reading strategies at their disposal, ranging from “hermeneutics” (Gadamer 1960/1990) via “discourse analysis” (Foucault 1969) up to “reading aloud” (Althusser & Balibar 1965/1970). Through such techniques, philosophers may distance themselves from everyday discourse and mainstream views – from *Gerede*, as Heidegger (1927/1986) once phrased it. Rather than acting as moderators or spokespersons of public opinion, they may function as suspicious minds, committed to developing “untimely” ethical, epistemological and ideological critique. The objective of this paper is to outline the methodological repertoire of a continental philosophy of technoscience as a diagnostic praxis.

## The *intentio obliqua*

In the current era of ELSA and RRI research, philosophers often work as “embedded” scholars in interdisciplinary settings, attending scientific lectures and meetings where research findings are discussed, but listening to such deliberations with a “different ear”. Rather than on the scientific content or technical details, they will focus on the ways in which the findings are presented, the signifiers that are used, the contrivances that are employed, the images that are projected, or the metaphors that are adopted. In other words: the axis of attention takes a quarter turn. Such lectures are followed from a slightly tilted, *oblique* perspective. Instead of on the object-pole (molecules, microbes, model organisms, etc.), the focus is rather on the subject-pole: the researcher or research team, the interaction between experimenters and their targets, “observing the observer”, as Bachelard (1938/1949, p. 13) once phrased it. They follow such lectures with evenly-posed attention (‘gleichschwebende Aufmerksamkeit’; Freud 1912/1943), and from a critical angle: a position which is comparable to how psychoanalysts keep track of the analysand’s discursive flow. At a certain point, somewhere in the stream of discourse, a specific image or concept may light up, triggering attention, catching the “philosophical ear”, so that a shift towards a more active, Socratic mode of listening is indicated, prompting questions and dialogue.

The *intentio obliqua* has a long history. Whereas science tends to focus on the object (the *intentio recta*), philosophy reflects on how this object is allowed to emerge (Breil 2011). Nicolai Hartmann (Hartmann 1935) argued that, whereas the “natural” direction of knowledge (the *intentio recta*, represented by natural science) is oriented towards the object under study, the “reflected” direction (*intentio obliqua*, i.e. knowledge reflecting upon itself) is typical for philosophy. The distinction goes back to medieval scholasticism. Thomas Aquinas already stated that, whereas human understanding is initially directed towards external reality, critical reflection on human understanding requires a change of perspective, an *intentio obliqua* (Schmidt 1966). By opting for an oblique perspective, a diagnostics of contemporary knowledge can be achieved: a critical assessment of the way contemporary life science allows living reality to emerge. This means that, rather than in protons, mitochondria or microbes, philosophers are interested in the λόγος–dimension: the words or signifiers that are actually used to bring such objects to the fore.

Bachelard once argued that, in terms of competence, philosophers have but one: “the competence of reading” (Bachelard 1948, p. 6). Not only in the sense that they are experienced or even voracious readers, but also because their reading is slow and interminable (Bachelard 1938/1949, p. 18), while the focus of attention is on the subject-pole rather than the object-pole of the knowledge relationship (on the microbiologist rather than on the microbe). How is the object isolated, dissected, brought to the fore and allowed to emerge? Research emerges as a dialectical process, and the focus is on how the object is prompted to reveal itself: on the practical, computational and discursive intricacies involved in conducting experiments. Thus, an oblique reading style entails an active form of reading, “with the pen at the ready” (“la plume à la main”), as Denis Diderot once phrased it. The axis of attention has taken a quarter turn.

Let me elucidate this with the help of some examples, taken from my own experience as a “practicing” philosopher. Since the 1990s, scientific discourse has produced a whole series of ‘-*omics*’ terms (‘genomics’, ‘proteomics’, ‘metabolomics’, ‘transcriptomics’ and so on). Such terms are closely connected with machinery, with big computers and high-throughput sequencing contrivances. They are the textual by-products of high-tech equipment, while their research targets are represented by a second series of signifiers (a parallel series of neologisms), ending with the suffix ‘–*ome*’ (the ‘genome’, the ‘proteome’, the ‘metabolome’, the ‘transcriptome’, the ‘connectome’, the ‘environome’, etc.). New labels containing an –*ome* or –*omics* component continue to show up, as new signifiers (composed along these lines) make their appearance. This grammar of –*omes* and –*omics* plays a performative role, rearranging rather than merely describing the evolving fields in question. Intriguing recent examples of –*omics* neologisms include the “unknome” (i.e. genes of unknown function, whose role scientists have not (yet) been able to identify); the “environome” or “exposome” (i.e. that part of external reality which can be sequenced by next-generation sequencing machines and deposited in electronic data-bases: the environment insofar as it becomes readable as molecular messages); and last but not least the “complexome” (i.e. the complex interactions between some of the –*omes* listed above).

The latter –*omics* target is studied by an unfolding research field called “complexomics”, a term which I encountered for the first time during a lecture on protons in mitochondrial research, so that my evenly-poised attention gave way to a Socratic stance. What does “complexomics” mean? Why was it coined? On the one hand, this signifier seems to underscore the bewildering complexity of life, allegedly beyond the grasp even of large-scale research consortia, notwithstanding the high-tech equipment (such as next-generation sequencing machines) and substantial funding arrangements available to them. But at the same time the label conveys the opposite idea, namely that (in the terabyte age) even the most complex interactions can be opened-up through digital analysis. In other words: the term *complexomics* conveys an attitude of modesty compensated by hubris. It is a “symptom”, a symptomatic phrase reflecting a basic ambivalence by both emphasising and obfuscating the complexities of living systems.

My second example comes from microbiology, where *gnotobiology* became a key signifier in contemporary microbiome research.^2^ Gnotobiology studies germ-free research animals, uncontaminated by micro-organisms (Gilbert & Neufeld 2014), although literally and originally the term refers to the study of animal models whose germs are fully known; − *gnoto*- comes from the Greek word γνῶσις (=knowledge). Gnotobiotic animals can only exist under gnotobiotic conditions: they spend their lives in germ-free bubbles inside laboratories, unable to survive in the open. In other words they are laboratory artefacts or bio-objects, units for experimental study and products of the “obsession” to eliminate microorganisms from human existence (Gilbert & Neufeld 2014, p. 1). The term reflects the technological prowess of scientists to construct and maintain germ-free, bio-conditioned dwellings. And whereas on the manifest level the focus is on reductionist control (stripping organisms of unnecessary ballast), a latent “germophobia”, a desire for catharsis and purification seems involved as well. Gnotobiology results from biology’s desire to purify and cleanse life in order to fully control it, by systematically reducing its messiness and increasing the transparency of living systems. Remember that the artificial human (the *homunculus*) produced in the laboratory of Goethe’s *Faust* (the primal scene or *Urszene* of the modern life sciences) likewise spent his life in a bubble, a gnotobiotic crystal vial (Goethe 1808/1910, 6884). The term γνῶσις has an alchemistic ring, moreover, as the creation of artificial life was originally an alchemistic dream. But also in modern science, knowledge is ideally achieved through purification and subtraction, which is the basic objective of this type of biomedical inquiry: living entities are stripped in a radical way of their messy surplus. One could even claim that all laboratory-based biology is heading in this direction, driven by the desire to become gnotobiology. To know microbes means to control them, while the ultimate aim is human organismal self-knowledge (γνῶθι σεαυτόν).

Whether discursive phenomena such as ‘complexomics’ and ‘gnotobiology’ are indeed symptomatic, telling and revealing (well chosen, in other words) must be validated by further analysis, but the tension between complexity and reductionism, or between purification and control, may claim an epistemological urgency which, I would argue, transcends these (more or less impromptu) examples. Thus, philosophers may overcome the concern already brought forward by Michel Serres (1972), namely that in the present era of disruptive change, exemplified by the emergence of large-scale, high-tech, trans-disciplinary research fields such as molecular biology, philosophy runs the risk of losing track, of becoming outdated and irrelevant. Philosophers may still play a vital role, Serres argues, provided they acquaint themselves with these newly emerging “oceans” of knowledge opened up by techno-science, from a position of close proximity, entering the tissues and capillaries of emerging research arenas as embedded scholars, addressing philosophical issues raised by these developments in close interaction with the scientists involved. Philosophy becomes “conceptual epidemiology”: analysing and assessing how techniques, vocabularies, concepts, metaphors and research practices spread through research fields worldwide, infecting and inflaming the tissues of the broader societal life-world as well. The oblique perspective sees research terminology as a by-product of technology and explicitly pays attention to how the technological ambiance conditions the emerging discursive world.

## From discursive symptom to philosopheme

*Complexomics* and *gnotobiology* are symptoms of a more basic discursive ambivalence, as we have argued. Although scientific research is allegedly awed by the complexities of living systems, it is also spurred on by the idea that the know-how and devices of technoscience have evolved sufficiently for life to become fully controllable and technologically reproducible. Terms such as *complexomics* and *gnotobiology* seem worthwhile to explore in detail because they provide a window into basic desires and tensions fuelling current life sciences discourse. Notably, they may tell us something about how basic concepts such as ‘life’, ‘nature’, ‘truth’ and ‘technology’ are envisioned and enframed in evolving research practices. As discursive events, neologisms such as *complexomics* may put us on the track of the guiding convictions at work in contemporary scientific discourse. Building on Hegel, but also on Bachelard, I will refer to these basic signifiers (‘life’, ‘nature’, ‘truth’, ‘technology’, etc.) as the *philosophemes* of scientific discourse (Hegel 1812/1986; Hegel 1832/1971; Bachelard 1949/1962, p. 7).

The signifier *complexomics* is a linguistic polymer, a collation of components, compressed into a neologism, but conveying crucial convictions of contemporary life sciences research. While –*ome* and –*omics* reflect the tendency towards molecularisation (bent on unravelling the basic molecular building blocks or barcodes of life: nucleotides, amino acids, proteins and so on), the prefix *complex-* rather refers to the complex interactions and emerging properties of living systems. In other words, complexomics *terminologically* combines a r*eductionist* view of life (focussed on analysis, on breaking-down the phenomena of life into basic molecular components) with a *holistic* view (life as an evolving synthesis of intimately entangled molecular processes). In other words, the term sublates (in a dialectical fashion) two (allegedly antithetical and incompatible) tendencies, namely reductionism (analysis) and holism (synthesis). It merges a focus on rewriting the barcodes of life with a focus on managing complexity, so that contemporary life sciences not only provide a high resolution understanding of life, but also allow the scientific gaze to leap towards a higher level complexity: beyond genetic determinism.

In other words, the signifier *complexomics* provides access into latent answers to some of the basic questions addressed in life sciences laboratories of today, such as: What is nature? What is life? What is technology? It reflects (in a highly condensed and symptomatic manner) basic conceptual categories. The term *complexomics* is symptomatic for how life sciences research is currently conducted. Hegel refers to these basic categories (these basic answers to questions such as What is nature? What is life? What is technology? etc.) as *philosophemes*. Moreover, all revolutions, in science no less than in world history, he argues, come about when the spirit (*Geist*) changes its basic categories.^3^ The term *complexomics* may be regarded as a symptom of a basic zeitgeist shift on the level of the philosophemes, a sublation away from genetic determinism (which focusses excessively on the explanatory power of biomolecular particles) towards complexity (seeing life as the end result of complex interactions between various dimensions of living systems, referred to as –*omes*). According to Hegel, philosophy’s vocation is to bring these basic categories to the fore, to discern and critically assess them. In other words, a philosophical reading entails a focus on symptomatic discursive events, reflecting basic shifts, tensions or inconsistencies on the level of the philosophemes: a symptomatic reading of the discourses produced by science.

## The core philosopheme of the natural sciences: what is nature?

Hegel’s claim that there is more philosophy in science than scientists are usually aware of, or willing to acknowledge (1830/1970, p. 11), remains a valid starting point. The oblique perspective proposes to bring this latent philosophical content to the surface by focussing on the symptomatic discursive events that point to shifts and tensions on the level of the *philosophemes*, the basic signifiers of scientific inquiry. During periods of transformation and disruption, such as we are witnessing today, exemplified by the emergence of big science and the advent of the terabyte age, the basic signifiers are under siege and in a state of flux, amenable to change. Genetic determinism (i.e. the claim that we basically are our genes) entails the conviction that the protein-coding genes on our genome are the program (in Hegelian terms: the “idea”) which realises itself in the life of the organism. This philosopheme (this basic understanding of what life essentially is), seeing genomes as programs and genes as the ultimate causal determinants of organismal phenomena, is now challenged and complemented by post-determinist approaches which emphasise complexity. This transition entails a shift of focus from linear causal relationships to emergent properties of complex systems, concurrent with similar shifts in other research areas and realms of culture. Philosophical intellectual labour consists in bringing to the fore (“herauspräparieren”: Hegel 1832/1971, p. 75) the philosophemes of scientific discourse, as condensations of the spirit of the time.

The most basic philosopheme of the life sciences concerns living nature and the most pressing philosophical objective is to determine what view of nature is at work in contemporary life science discourse. As a practicing philosopher, reading and listening to scientific discourse from an oblique perspective, it often strikes me that nature is not only studied in laboratories, but also represented as a laboratory (Zwart et al. 2015). From the perspective of post-genomics and synthetic biology, nature as such emerges as an outdoors laboratory of immense complexity and proportions, where a plethora of interminable experiments are being conducted (known as evolution), while in man-made laboratories these natural experiments are replicated, plagiarised and modified. Rather than being a mere metaphor for nature, it entails an ontological claim: nature really is a laboratory, from the viewpoint of contemporary science.

This idea is not completely new, of course and its genealogical origins can be traced at least as far back as the second half of the eighteenth century, when the first laboratories came into being. Denis Diderot (1754/1983), for instance, already saw nature as a laboratory or test-bed where (in the course of evolution) countless varieties of species, limbs and organs had been systematically probed and tested.^4^ And laboratory animals are likewise described by Diderot as living, organic “laboratories” whose “sensibility” is opened-up through active modification.

In my experience this has become the dominant view, notably in emerging research arenas such as synthetic biology. George Church, for instance, one of its key protagonists, argues that evolution happens both in nature and in laboratories: environments which mirror one another (Church & Regis 2013). Likewise, according to Elowitz and Leibler (2000), a cell is basically a “laboratory” which allows scientists to “tinker” with life and to explore questions of biomolecular design in depth. As Meyerhoff and Lietman (2009) argue: from a biomolecular perspective, “all the world’s a laboratory”. Microbes and viruses are constantly conducting experiments in the wild, while life sciences laboratories aim to mimic natural laboratories as closely as possible (Benyus 1997). Purportedly, there is no real difference between natural biotechnology (developed and used by microbes and other organisms in the course of evolution) and artificial biotechnology (employed in man-made laboratories around the globe). This way of seeing cells and organisms, or even our own bodies, has become ubiquitous, even self-evident in science. Therefore, while listening (with evenly-poised attention) to lectures about proteins, mitochondria and microbes, the laboratory view of nature is what philosophers may want to grasp. Not in the sense of counting the number of times the phrase “nature is a laboratory” is actually used, but rather by studying the terminologies which (often in subtle and inferential ways) suggest, convey and reinforce this view. Thus, the oblique perspective focuses on the technical way in which proteins, cells and molecular processes are depicted. The view of nature as a laboratory has become the inescapable metaphysical horizon of contemporary scientific research: its most pertinent philosopheme.

This line of thinking concurs with how Martin Heidegger studied modern science. In *Wissenschaft und Besinnung* (“Science and Reflection”), for instance, he claims that contemporary science evidently developed an unprecedented sway over reality, acquiring a tremendous impact on how reality presents itself to us (Heidegger 1953/1954). To a considerable extent, the term ‘nature’ basically refers to nature as it is brought forward and presented through scientific research. As a key example, Heidegger refers to elementary particle physics, whose ‘objects’ (i.e. subatomic elementary particles) are forced to reveal themselves via hyper-powerful machines (particle colliders), forcing them into existence as it were, determining how and when they may become ‘visible’ to us, − insofar as ‘visibility’ still has any meaning here at all, for ultimately these particles are bound to remain intractable ‘objects’: spectral, transitory and evaporating phenomena, on the verge of appearing and disappearing. We only ‘see’ them at their moment of annihilation.

This inevitably raises the question What is nature? Ultimately, Heidegger argues, nature cannot be identified with the ‘objectivity’ produced by science. Nature (φύσις) keeps escaping us and is bound to remain something other, something Real: nature as that which continues to withdraw itself, which refuses to become completely identifiable and compatible with the techno-scientific way of making reality visible; nature as that which flashes up, in a recalcitrant manner, in the folds and margins of the scientific representation of the world. Furthermore, are we humans really in control? According to Heidegger, another force seems to determine the whole process: technology as such, expanding and proliferating, dragging research along. Something other than human inquisitiveness seems to hold sway over this endeavour. According to Heidegger, this hidden power will remain concealed from us if we fail to really question the fundamental terms at work in contemporary, technology-driven science, − first and foremost: the term nature.

The view of nature as an immense outdoors in vivo laboratory discloses nature in an outspokenly technical way. As long as we merely adopt this latent view, rather than questioning it, it will pervade scientific discourse up to the point of becoming self-evident and seemingly unquestionable, so that not only science but also philosophy of science will fall victim to its sway. Researchers will be put to work in research laboratories to confirm its truth. It is only by questioning the grounding dominating terms, the philosophemes, via an oblique perspective, that we can distance ourselves from this obfuscating *termino-logical* ambiance, which blinds us to other possible answers concerning the question What is nature? The conception of nature is the “philosopheme of philosophemes”, the basic metaphysical conviction guiding scientific research, affecting experimental and discursive practices as an epistemological pandemic, pre-structuring the way in which derivative terms and concepts are defined.

## The subject-pole of the knowledge relationship

Besides neologisms referring to the object-pole (such as *complexome*), discursive phenomena associated with the subject-pole may likewise arouse attention, such as the current endemic focus on (or even obsession with) “misconduct” and “integrity” in research. A possible explanation for the current prevalence of such labels could be to regard them as symptomatic compensations in response to the decentralisation, anonymisation and collectivisation of the scientific “subject”, of the researchers themselves. The focus on misconduct could reflect growing tensions between traditional research ethics (focused on autonomous, responsible researchers, conducting researcher-driven experiments, publishing results as single authors or small teams) and the emerging trend towards large-scale research consortia, which includes automation and multiple authorship, resulting in a marginalisation of the scientific individual (Zwart 2008).

Habermas (1968/1973), Serres (1972) and others already argued that research is becoming large-scale and hyperactive. This has repercussion not only for the object-pole of the scientific endeavour (where ‘objects’ are becoming increasingly artificial, modifiable and technologically reproducible with the help of high-tech research equipment), but also for the subject-pole. Increasingly, research is transferred to machines and, according to Habermas (1968/1973), this not only applies to the monotonous handiwork of science, but also to brain work, to thinking as such. Humans eventually become mere operators, highly dependent on their equipment. They themselves increasingly become components within complicated networks of machines: “living accessories” in a machine park.^5^

As a consequence, individual conscience and self-consciousness become increasingly irrelevant, Habermas argues and research even immunises itself against ‘generalised’ self-consciousness, i.e. philosophical reflection. In other words, for Habermas, the trend is toward the marginalisation and instrumentalisation of the subject, whose activities become automated and overregulated. But as individuals will never completely coincide with their pre-formatted roles, they will increasingly become a source of confusion and deceit, or even potential frauds. Serres (1972) likewise argues that, in research fields such as molecular biology, researchers are decentred by automation and laboratory equipment, and outcompeted by computers and robotics. They may even become a superfluous burden, a source of error or misconduct. And this is already happening. Oblique explorations bring the broader landscape into view which gives rise to the debate (currently in vogue, especially among research managers, university boards and funding agencies) on research integrity and misconduct. In other words, before providing a (technical) solution (a “therapy”, in the form of regulations, etc.), the oblique approach will first of all explore the emerging integrity challenges which researchers are actually facing (“diagnostics”).

To overcome this growing resistance and immunisation against reflection, Habermas (1968/1973) endorses a psychoanalytical approach, regarding psychoanalysis as the most significant surviving representative of the critical philosophical tradition, urging us to see emerging tensions and conflicts as symptoms of more basic ambivalences, or even pathologies. Moreover, rather than accepting normalisation and collectivisation, psychoanalysis persistently focuses on individual dramas and biographies, summoning researchers to work through and critically assess their experiences, for instance with regard to ‘object choice’.^6^ In the next section I will explain how Lacanian psychoanalysis, notably Lacan’s theorem of the four discourses (a crucial component of a contemporary psychoanalysis of science), further elucidates the oblique perspective as a research strategy.

## The oblique perspective and the four discourses

The key objective of the *intentio obliqua* is to bring to the fore (“herauspräparieren”) and critically assess the basic *philosophemes* (premises, imperatives, performative signifiers) of contemporary scientific discourse. Although they often remain inarticulate, they provide pervasive guidance. Building on Lacan (1969-1970/1991), I will refer to these basic signifiers (this conceptual-terminological base) as S_1_, and the discourse that is fuelled and spurred on by them as S_2_. The most basic philosopheme of contemporary life sciences discourse, as we have seen, involves the signifier “nature” and can be summarised in shorthand as “nature = a laboratory”. This idea, although hardly ever articulated and discussed explicitly, pre-structures emerging life sciences discourse. In terms of Lacan’s symbolic algebra, the relationship between mainstream life sciences discourse (S_2_) and its guiding premises (S_1_) can be represented as: S_2_/S_1_, so that normal scientific discourse (S_2_, i.e. “physics” in the general sense of the term: the experimental study of nature, φύσις) builds on, but is at the same time literally barred from explicitly addressing its basic metaphysical premises (S_1_).

In principle these basic philosophemes and imperatives may be presented in a top-down, apodictic, authoritative, *ex cathedra* fashion. In that case, S_1_ is posited at the top-side of the bar, resulting in what Jacques Lacan (1969-1970/1991) refers to as the Master’s discourse, exemplified by the writings of an authoritative author (*Aristoteles dixit*). The Master is regarded as infallible, his claims and premises provide guidance to readers (students spelling his oeuvre) and the Master’s name serves as index of truth. Any uncertainties or doubts on the part of the Master, which must have troubled him during his life-time as a practicing philosopher (in Lacanian algebra: *$*, i.e. the researcher as a divided subject), are disavowed and suppressed, that is: placed beneath the bar: S_1_/*$*. The discourse of the Master addresses a scholastic readership. In contemporary philosophy, this role is conducted by author studies experts, the custodian of the Master’s oeuvre (S_2_). Such experts are put to work, as discursive servants and servile subjects, to explain and defend the integrity and authority of the Master’s corpus (body of writing), resulting in a particular kind of expertise or expert discourse: S_2_ (which refers both to the discourse as such and to the subjects who generate it). Such university experts focus their attention on the cardinal signifiers of the Master’s oeuvre, which (due to their opacity and intricacy) often prove a source of frustration, but of intellectual jouissance as well. Scholars may be drawn towards one particular ungraspable, unfathomable, “impossible” concept, functioning as the intractable, inexorable “object *a*” of the Master’s discourse, as Lacan phrases it. Aristotle’s concept of the Divine Intellect which thinks itself, for instance, may stand as an example: God conceived as “thinking thinking thinking” (νόησις νοήσεως νόησις, Metaphysics Λ, 1074b), while a more mundane example would be the *causa finalis* (the cause that works backwards in time, so that something is caused by something else which is not yet realised or brought about itself, a logical impossibility for modern science). Such “impossible” key concepts result in a plethora of books and papers and offer windows into the oeuvre as a whole. This combination of frustration and jouissance, circling around the object *a*, and resulting in a particular type of (servile, apologetic) discourse, is regarded as the by-product of the Master’s discourse.

With the help of four key symbols (S_1_, S_2_, *$* and *a*), Lacan distinguishes four basic types of discourse. The four basic symbols may be inserted as “variables” (in a fixed sequence: *$*, S_1_, S_2_ and *a*) in four positions in a rotating, revolving, quadruped scheme:

In the case of the Master’s discourse, this results in the following scheme:

The Master (in the upper-left position of the agent) is an acknowledged, allegedly infallible, authoritative voice, as we have seen. Uncertainties, disappointments and doubts to which the Master as a real, craving individual (*$*) may have fallen victim in real life, are decidedly left out of the picture, suppressed beneath the bar (S_1_/*$*). Masters address disciples (in the upper-right position, as recipients of the message) and produce a particular type of discourse, immersed in contemplation, metaphysics and basic geometry. Plato and Aristotle may count as paradigmatic examples of Master-thinkers or gentlemen-philosophers. They contemplate nature as a harmonious spherical whole: a κόσμος, and hardly concern themselves with concrete interactions with real nature (Zwart 2009). They develop a platonic view of nature.

As Lacan explains, this type of Master’s discourse (dominated by S_1_) contrasts with the discourse of the servant, whose knowledge is basically know-how (“savoir-faire”, Lacan 1969-1970/1991, p. 21). The Master (the gentleman-philosopher) is initially in control. He appropriates the servant’s practical knowledge and transforms it into abstract knowledge (ἐπιστήμη, θεωρία), for instance: Euclidean geometry. Lacan points to the dialogue between Socrates and the slave Meno, where Socrates acts as a benevolent gentleman-teacher, granting the illiterate slave a crash course into Euclidean geometry, only to discover that the slave already knows his geometry, albeit in a practical, hands-on way. Theoretical knowledge (Euclidean geometry, ἐπιστήμη) has been appropriated by the Master, who transforms it into apodictic, deductive knowledge and now purports to give it back, as a gift, in the form of education (Lacan 1969-1970/1991, p. 22).

But in the end, the practical knowledge of the servants will prove much more powerful and effective compared to the lofty contemplations of the Masters who, instead of really interacting with and transforming nature, rather develop a worldview, i.e. an imaginary vision of nature (as a spherical, harmonious whole, a κόσμος). Eventually, the supremacy of the Master (S_1_) will by subverted by the practical know-how of the servant (S_2_), so that in the end S_2_ will come to occupy (usurp) the upper-left position as agent. The power of the Master is subverted (S_2_/S_1_), the Master’s voice suppressed and the scheme takes a quarter turn to the left.

Hegel’s dialectics of Master and Servant, developed in his Phenomenology of the Spirit (Hegel 1807/1973) may elucidate this inevitable dialectical turn. Initially, the Servant acknowledges the supremacy of the Master. Instead of challenging the latter’s authority, the Servant willingly relinquishes his own autonomy, opting for an attitude of devotion and servitude. Such servants are put to work, in the interest of the Master. Rather than aspiring to become Masters themselves, which would lead to competition and warfare, they accept a subordinate position of dependency. This type of servitude produces a particular form of jouissance, for the servant guards the Master’s truth. Inevitably, however, a dialectal dynamics unfolds, which eventually subverts the situation in the sense that the discourse of the Master becomes increasingly dependent on the work of the servants. They become increasingly skilful, first of all as custodians and interpreters of the Master’s founding gestures.

But the emancipation of the servants does not stop there. Rather, instead of relying on the signifiers coined by the Master to understand nature, the servants will explore and interact with nature more directly. Increasingly, the Master’s apodictic views are suppressed (pushed beneath the bar), as servants rely on hands-on, practical interactions with nature, developing powerful tools to manipulate and manage natural objects more effectively: the birth of the experimental method. Exegesis increasingly gives way to experimental work (manipulating and quantifying nature). Via skills and know-how, the servants assume mastery over the situation. They become scientists, scientific agents (S_2_ in the upper-left position), while the meta-physical pontifications of the Master becomes a superfluous burden, so that the power relationship becomes subverted, and a new type of discourse emerges, to which Lacan refers as the university discourse:

Now the Master no longer addresses the Servant explicitly. The Master’s imperatives are disavowed, repressed and pushed beneath the bar. The former servants have emancipated themselves: they have become scientific experts, addressing nature on their own accord. They focus their attention on a particular object, however, a particular problem or process, a particular molecule or model organism: a particular object of choice (*a*). Rather than studying living nature as a whole, a κόσμος, nature becomes condensed and compressed into a particularly intriguing but highly demanding entity (*a*). Although initially the scientists (S_2_) seem in control of the situation, eventually the unfathomable object may prove a demanding, addictive, toxic lure. Instead of the expert being in control (manipulating the object) it is the other way around: the object becomes the active force, drawing the researcher towards it.

Take for example John Sulston’s research on the (hermaphrodite) nematode worm C-Elegans. In his auto-biography he explains how he “first met the worm” (Sulston & Ferry 2003, p. 17) in 1969 in the Laboratory of Molecular Biology in Cambridge (UK): a tiny, self-fertilising species, a millimetre long, while Sulston was given a metre of bench space to work on it, a labour which he later continued in San Diego. As a scientific monk he spent many years tracing, with the help of a special microscope, the development of all the 959 cells of the nematode’s body, and would eventually be awarded the Nobel Prize for this. But for many other researchers, the object of choice will rather prove a source of frustration, resulting in various symptoms, from workaholism via burn-out up to fraud (*$*). Rather than experiencing gratification and success, scientific subjects will often find themselves hopelessly chained to and drained by their inexorable object *a*.

This dialectical schema may also help to understand the changing relationships between philosophy and science. Philosophy no longer occupies the position of the Master, as it did during previous epochs, when metaphysics was still in vogue (S_1_ as agent). The former servants acquired agency via experimental, hands-on, technology-based research (‘laboratory’ literally means workshop), actively interacting with their research objects. Scientists develop increasingly effective lab tools to generate robust knowledge and refurbish nature. The contemplating gentleman is dethroned, and metaphysics no longer provides apodictic guidance. Metaphysics is marginalised, becomes a research field *in statu moriendi*, and yet it is still there, occupying the position of the (suppressed, latent, disavowed) truth of scientific discourse (S_1_ below the bar).

In the Introduction to his Philosophy of Nature, Hegel (1830/1970) deplores that metaphysics, the Master’s discourse par excellence, has fallen into disrepute. Metaphysics has been replaced and subverted by insights produced by natural science. A field of knowledge which once aspired supremacy over other (more practical and reality-oriented) fields has now fallen silent.^7^ But rather than becoming obsolete, philosophy finds itself in a new position (1818/1970, p. 402). The era of metaphysics did not end with the rise of laboratory science, Hegel argues, but the focus of attention must now shift to the implicit metaphysics at work in scientific discourse (S_1_, the basic premises, pushed beneath the bar, as the terminological unconscious of science). By taking up this challenge, a new dawn (‘*Morgenröte*’) may set in. Philosophers may question and critically assess the latent but guiding philosophemes (S_1_) of science. Rather than being delisted from the agenda, the metaphysical question “What is nature?” proves inescapable. We are both drawn to and repelled by this question: difficult to answer, but impossible not to ask (Hegel 1830/1970). We simply cannot ignore this basic philosopheme of science.^8^ Scientific research (S_2_) it not a purely technical or empirical endeavor, but entails a profound, yet tacit understanding of nature (S_1_), an inspiring truth, which can and should be brought to the fore and critically examined by philosophy. Science is adrift, moreover. We are in the middle of a scientific revolution, so that the philosophemes of science are becoming fundamentally questionable, are being drastically redefined (S_1_ → S_1_).

Experimental researchers (S_2_) as agents (upper-left position) focus their attention on various kinds of objects as targets of their *cupido sciendi*, their will to know. Laboratory objects (a particular type of microbe, virus or protein or a particular model organism) function as the intractable entity (object *a* in the upper-right position) which drains their intellectual energy, time and resources, but continues to escape them, for instance because initial results cannot be replicated. In normal science, the laboratory expert (S_2_) as agent seems firmly in control, but in real laboratory life, scientists may fall victim to the situation, become trapped by the inexorable object *a*, on which a whole life-time may be wasted. Due to frustrations awaiting them, scientists become tormented subjects (*$* in the lower-right position), with discontent and doubt as by-products of experimental research.

## The oblique perspective as the discourse of the analyst

What mode of discourse will philosophy generate, looking at and listening to science from an oblique perspective? Rather than apodictic deductions (as in the Master’s discourse), the oblique perspective involves hard labour, with philosophers working their way through the archives, the multiple and interminable discourses of science. Philosophy becomes research, but in its own (oblique) way. The focus is neither on the oeuvre of the Master (as in author studies), nor on developing a specific type of expertise (such as health law or bioethics, which concur, in terms of discursive structure, with university discourse), but rather on the ways in which life science research is enacted and life sciences discourse is framed.

Some instances of philosophical inquiry may reflect what Lacan refers to as university discourse, namely when philosophers aspire to develop specialized expert knowledge, as ethical experts for instance, applying basic sets of principles or argumentative skills to cases. Such experts serve as ethical engineers. Mainstream applied bioethics reflects the university mode of discourse when ethical expertise basically consists in a particular kind of literacy and fluency concerning a particular ethical grammar, developed for analysing and addressing moral dilemmas in preformatted ways. Other philosophers may become the custodians of an oeuvre, of the intellectual legacy of a deceased author, which threatens to become a dead letter (discursive “litter” as it were) and therefore has to be reinterpreted, reanimated. In this discursive mode, the experts (S_2_) relinquish the ideal of becoming genuine philosophers themselves (addressing issues emerging in science and society in an active manner, moving beyond established discourse, perhaps experiencing the euphoria of a truth event), but rather settle for the more moderate joys of the disciple, guarding the Master’s treasures against vulgarisation.

Philosophers may also opt for what Lacan refers to as the *hysteric’s discourse*. In this case, the tormented, divided subject (in Lacanian algebra: *$*) emphatically takes the floor as agent, raising a voice of societal protest. This type of discourse figures prominently in societal debates on science and technology, where philosophers may become activists, challenging the voice of authority, the authoritative Other as the recipient of the message (S_1_ in the upper-right position):

In his book *Critique of Cynical Reason*, Peter Sloterdijk (1983) endorses this type of discourse as a genuine philosophical position, by tracing its genealogy, which takes us back to the ancient Cynics: a boisterous tradition relying on provocative gestures and dramatic, ludicrous or scandalous interventions, a bold, impertinent, popular, gay, practical, provocative, theatrical and grotesque style of moral critique (Zwart 2016).

An oblique perspective, however, confronted with the (often boisterous and passionate) interactions between *$* and S_1_, will spur these activists towards self-reflection. What is really driving their protest (often directed at very specific targets), what kind of uneasiness or desire is at work beneath the bar, pointing beyond the issue at hand perhaps, towards a more basic discontent in science, or in civilisation even? What do these activists really want? By asking such questions, philosophers have already entered a different type of discourse, namely the discourse of the analyst.

Although philosophers may play various roles and may function as Master (the philosopher as guru: S_1_ as agent), as experts (in author studies or applied ethics: S_2_ as agent), or as activist (*$* as agent), a fourth type of discourse is more recommendable and concurrent with the oblique perspective, namely the *discourse of the analyst*, a paradoxical term, since (ideally) the analyst is the one who does not speak, but rather listens, with evenly-poised attention. For this type of discourse to work, the philosopher’s expertise and knowledge (S_2_) must be suspended, placed beneath the bar (lower-right position), at least temporarily: a position known as learned ignorance (*docta ignorantia*). But precisely because of this intellectual self-constraint, this willingness to bracket established philosophical views concerning life, science, nature and technology (ἐποχή), the floor is open to other voices, to experiences of practicing researchers, driven by a scientific will to know (their *cupido sciendi*). Thus, the ultimate target of desire, referred to by Lacan as the inexorable object a, comes into view, occupying the position of agent: triggering, commanding and frustrating the scientists’ interminable work. This object challenges the scientists’ prowess and arouses their desire, but continues to escape them, so that they emerge as tormented subjects (*$* in the upper-right position).

This type of discourse builds on the tradition inaugurated by Socrates, and the oblique approach is quite compatible with his ethos, bent on transforming seemingly every-day settings (lectures, discussions, readings, meetings, site-visits, etc.) into philosophical laboratories, where the philosophemes of contemporary discourse can be articulated and examined:

Psychoanalysis is not a science, but a discursive practice prompting self-reflection. What is it that researchers find so fascinating about their object *a*? Why do they waste the most fruitful years of their life on this alluring entity, why do they consider it the panacea or missing link? Oblique philosophy basically entails embedded dialogue, however and philosophical interpretations and assessments are only valid and effective insofar as they provoke further deliberations and reflections on the part of the scientific subjects themselves (i.e. mutual learning).

A risk involved in this type of discourse is that, in the end, the analyst is mistaken for a Master, the author of an opaque, authoritative and apodictic discourse, giving rise to discursive servitude (S_1_ in the lower-right position), as happened with authors such as Freud and Lacan, so that their followers fall into the trap of posing as servile, apologetic “experts” of an oeuvre, rather than as active philosophers themselves, oriented towards assessing and questioning the emerging discourses of technoscience from an oblique perspective. But in the current era, where philosophical reflection has become a collective and distributed endeavour, such a scenario has become less likely.

This does not imply that philosophers should engage in the discourse of the analyst continuously. In the unfolding process they may switch to other types of discourse, opt for other discursive modes, temporarily acting as author studies expert, for instance or ethics expert, or social activist, but the discourse of the analyst, concurring with the oblique perspective, allows us to discern the strengths and weaknesses, opportunities and traps of these discursive options. As Hegel phrased it, rather than being the first to speak (as agent), philosophers spread their wings at dusk, as owls of Minerva, when other types of discourse have already thrived, when other agents (S_1_, S_2_, *$*) have already spoken. The philosopher’s intellectual labour consists in reading and listening with evenly-poised attention to how others have already responded to the situation. Rather than opting for expertise, activism or pontification, oblique philosophers point to discursive symptoms, ambiguities, blind spots and contradictions that reflect the philosophemes adrift. The starting point is that we no longer know what nature, life, truth, technology, etc. really is. Such issues emerge in the context of a critical dialogue, a living oblique laboratory, a mutual learning exercise.

Thus, an oblique (symptomatic) reading of contemporary life sciences (as an interminable flow of university discourse) will focus on the symptoms that allow philosophical intentionality to shift from scientific discourse as such (S_2_) to the philosophemes that actually guide and structure it (S_1_), but also to the tormented subjects (*$*) who aspire to adhere to normalised discourse and its imperatives, but experience challenging obstacles and inhibitions in their interactions with their object of desire (*a*). Such researchers may even be tempted to commit “misconduct” in order to maintain a semblance of normality and performativity. In order to detect and disclose the philosophemes (S_1_), specific signifiers are singled out as especially relevant. Building on the etymology of λόγος (Heidegger 1951/1954), an oblique reading (*lectio*) tends to be selective, so that *lectio* becomes *selectio* and attention becomes fixed on specific, revelatory terms, reflecting in a symptomatic manner the shifting *philosophemes*. But it is via the discourse of the (apparently normalised, but actually challenged and tormented) scientific subjects that these philosophemes are disclosed (S_2_ → *$* → S_1_).

## Discourse-, subject- or object-centred?

Via established scientific discourse (S_2_, the flow of scientific signifiers) and the speech acts of challenged, tormented scientists (*$*), the oblique perspective exposes the philosophemes of science (S_1_), i.e. the imperatives that guide researchers towards the object of their *cupido sciendi* (*a*). In Lacanian algebra normal scientific discourse can be represented as (S_2_ ◊ *a*), where S_2_ refers to the discourse of university experts (as agents) while *a* represents the target of their will to know, and the lozenge or *poinçon* (◊) stands for laboratory contrivances, for instance optic devices such microscopes, enabling experimenters to zoom out (<) or in (>), bringing the object into view while keeping their distance. This suggests that the scientific agent is firmly in control, but in reality researchers may fall victim to the situation and revert to the position of the tormented, desiring subject (S_2_ → *$*) while the allegedly normalised object may prove an ungraspable, inexorable, disturbing factor, putting the subject out of balance, so that the standard formula (S_2_ ◊ *a*) actually is a cover-up, a façade for what really should be represented as (*$* ◊ *a*), − a Lacanian equation known as the matheme of desire.

The basic tendency in scientific research is towards *anonymisation* and *normalisation* of the scientific subject. Researchers are expected to relinquish their “subjective” fascinations, interests and desires and to become mainstreamed contributors (*$* → S_2_), a tendency which is reinforced by automation and high-tech research contrivances (represented by the lozenge), but also by the use of technical terms, standardised formats and formulaic phrases in academic writing (S_2_). Research has become large-scale teamwork conducted by consortia employing big machines and resulting in multiple author output, where hundreds of researchers may be listed as author, in alphabetic order. Thus, the technification and standardisation of the object is paralleled by technical forms of authorship (Foucault 1969/1994), where author names are basically used to facilitate *retrieval* (as search terms) or *quality assessment* (of research groups) or as shorthand for theorems, syndromes or instruments (*eponymy*). Authorship attribution is increasingly becoming a device for facilitating the production, storage, circulation and retrieval of texts (preferably in electronic formats) or for detecting and penalising misconduct.

That the basic attitude of scientific research is discourse-centred rather than subject-centred was already emphasised by Nietzsche in *Dawn of Day* (1881/1980, § 547). Until recently, he argues, the scientist was a genius, a privileged individual expected to solve big riddles in a single, brilliant stroke. In contemporary science, however, such forms of ego-centricity have clearly become untenable. Research is teamwork, employing anonymous (often early stage) researchers who are closely supervised, while most of the actual work is effectively carried out by machines, and Nietzsche foresaw this when he claimed that in the future, the role of the scientific individual would become increasingly marginalised: “What do I matter?” should be written over the scientist’s door.^9^ In his essay on the death of the author (already cited), Foucault (1969/1994) endorses this view. What does it matter who is speaking? In this indifference towards individuality, Foucault argues, resides the fundamental ethos of contemporary scientific discourse.^10^ The core conviction that research findings should be replicable already implies that researchers should be replaceable. Scientific discourse is framed as an anonymous and interminable practice.

To some extent, this ethos has been there from the very beginning. Heraclitus already urged his audience not to pay attention to him (as a person) but rather, via him, to reason as such.^11^ Ideally, λόγος (reason, language, discourse) speaks. In contemporary scientific discourse this imperative seems very much alive. While browsing through the scientific literature, we read discourse rather than authors. In science, ‘it’ speaks, resulting in a continuous, interminable, proliferating flow of anonymous words without authors (S_2_). To single out one particular author (or even a small number of authors), in the context of Nobel Prize award procedures for instance, seems increasingly unfeasible and unfair (Zwart 2010).

Thus, the subject-pole of the knowledge dynamics is exposed to similar processes of purification and standardisation as the object-pole. The subject is effectively decentred, depersonalised and emptied of its ideological, subjective content, through training and socialisation, but also via automation and laboratory equipment. The subject is cleansed of its sociocultural heritage of ideas and associations concerning ‘nature’, ‘life’, ‘embodiment’ etc. (Bachelard 1938/1947), of its traditional Bildung, so that ideally a reliable, depersonalised and highly functional subject remains, dwelling in laboratories, smoothly interacting with (and increasingly replaceable by) machines: a subject without psychic depth: a kenotic subject (Zwart 2016).^12^

Yet, this can never be fully achieved, due to the recalcitrance of the research targets involved. Individuals will eventually prove unable to completely live up to the methodological imperatives proclaimed by the demanding superego of Big Science (S_1_). They are tormented by desire, mistrusted as potential frauds and hyper-actively obsessed with their research object of choice (*$* ◊ *a*), filtering out anything else as noise. They become introverts, stubbornly refusing to displace their intentionality to something else, or to be replaced themselves (as this would imply separation from their laboratory object). This may raise intriguing questions, such as: why has this particular object (this particular molecule, microbe or model organism) become such an object of desire, such a fetish: the sole and life-long target of the scientist’s *cupido sciendi*? By addressing such questions, however, we have already opted for an oblique style of reading, a change of perspective and the focus of attention reverts from ‘context of justification’ to ‘context of discovery’, analysing concrete subjects who, in concrete research settings, face demanding objects (*a*). Thus, the oblique perspective develops an interest in science biographies or autobiographies, “case histories” which report in detail how researchers not only manipulate and purify their object, but also are addressed and edified (as well as tormented and frustrated) by these demanding entities.

An intriguing example is DNA-researcher Maurice Wilkins (2003/2005) who, in his memoirs, describes his obsessive efforts to produce pure, undiluted strands of DNA, until at a certain point his DNA is so “excellent” that it is shouting at him, “Look how regular I am!” (p. 124). The experience of DNA as something which speaks out to a researcher is also conveyed by Watson in the movie *Life Story* (Jackson 1987), based on autobiographical reports, where he exclaims, after Wilkins has handed him Rosalind Franklin’s infamous photograph 51: “I could not believe my eyes; it was just sitting there, yelling out information, like a speak your weight machine” (Zwart 2015). In other words, the replaceable expert of normal science (S_2_) is actually a desiring subject (*$*), confronted with a demanding object (*$* ◊ *a*). The oblique perspective concurs with the discourse of the analyst, focussing on the object *a* (in the upper-left position of agent) as something which actively addresses and enforces itself upon the subject (in the upper-left position as recipient).

While continental philosophers often act as custodians of a Master’s discourse (as experts of an oeuvre), the oblique perspective entails a different role, analysing the dialectical interaction between tormented researchers (*$*) and their objects of desire (*a*). And instead of opting for a top-down, metaphysical approach, philosophers read and reread the scientific files, the avalanche of papers produced by laboratories worldwide, with evenly-poised attention, from a tilted, oblique perspective, using revelatory signifiers (*complexomics*, *gnotobiology*, etc.) as discursive symptoms, probing them with the help of a diagnostic reflex hammer, a plessor, a stethoscope, a magnifying-glass.

It is via discourse that the scientific object comes into focus. In post-phenomenology (Verbeek 2000/2005) and object-oriented ontology (Harman 2011) the question has been raised whether philosophy, by focussing on speaking subjects (on discourse), neglects and obscures the things, the objects. Is the oblique perspective a retreat into purely linguistic terrain? As Coeckelbergh (2015) emphasises, phrases such as “language or technology” or “subject or object” are misleading. It is via the discourse of the tormented scientific researcher that the intractable “object *a*” comes into view (*$* ◊ *a*). In normal science, researchers prefer to work with normalised, standardised objects of research (molecules, microbes, model organisms, etc.). Although they once were challenging targets, they are now domesticated and transformed into a research tool, a fully controllable laboratory device. Research targets such as gnotobiotic model organisms become reproducible units within techno-scientific arrangements. Model organisms (from C. elegans down to bacteriophages) are products of laboratory settings. And “-*omics*” entities (genomes, metabolomes, transcriptomes, etc.) are likewise intimately connected with technology. They are hyper-technical “objects” and can only exist in a highly specialised technological ambiance. Yet, in the folds and margins of normalised and established research practices, unexpected findings may point to the presence of disruptive factors: the intrusion of a treacherous object *a*, a factor X, inciting suspicion and evoking desire. The oblique perspective implies that the object-pole comes into view via the discourse of the scientific expert. It is not our decision to study genomes, amino acids or synthetic cells. Rather, the intentionality of the oblique perspective is determined by the scientific research practices under study. Their objects (genes, proteins, genomes, etc.) become our objects as well, approaching them from an obliquely perspective.

## Footnotes

1 Das was ist zu begreifen, ist die Aufgabe der Philosophie … [Sie ist] ihre Zeit in Gedanken erfasst (Georg Wilhelm Friedrich Hegel 1821/1970, p. 26)

2 The term was already introduced in the 1960s (Luckey 1963) and its genealogy stretches as far back as the work of Pasteur and Metchnikoff.

3 “Alle Revolutionen, in den Wissenschaften nicht weniger als in der Weltgeschichte, kommen nur daher, dass der Geist … seine Kategorien geändert hat” (Hegel 1830/1970, 9, § 246, Zusatz, p. 21).

4
*Letter to Duclos*, October 10, 1765.

5 This even applies to the (digital) humanities, by the way, where close reading gives way to “distant reading” (Herrnstein Smith 2016).

6 While endorsing psychoanalysis, Habermas (1968/1973) vehemently rejects the positions of Nietzsche: the self-annihilation and abnegation of reflection as something superfluous, self-deceptive and meaningless.

7 “Diese Morgenröte begrüße ich, rufe ich an, mit ihm nur habe ich es zu tun” (Hegel 1818/1970, 10, p. 403).

8 Was ist die Natur? Wir finden die Natur als ein Rätsel und Problem vor uns, das wir ebenso aufzulösen uns getrieben fühlen, als wir davon abgestoßen werden… Wir sammeln Kenntnisse über die mannigfaltigen Gestaltungen und Gesetze der Natur; dies geht in ein unendliches Detail hinaus, hinauf, hinunter, hinein; und eben weil kein Ende darin abzusehen ist, so befriedigt uns dieses Verfahren nicht. Und in allem diesem Reichtum der Erkenntnis kann uns die Frage von neuem kommen oder erst entstehen: was ist die Natur? Sie bleibt ein Problem (Hegel 1830/1970, p. 12).

9 “Daraus ergibt sich, daß im Großen und Ganzen die Wissenschaft … mit einer höheren und großmütigeren Grundempfindung fürderhin getrieben werden muß. ‘Was liegt an mir!’ – steht über der Tür des künftigen Denkers” (Nietzsche 1881/1980, § 547).

10
*Qu’importe qui parle? En cette indifférence s’affirme le principe éthique, de plus fundamental peut-être, de l’écriture contemporaine* (Foucault 1994, 789); Cf. Hegel: “Weil … der Anteil, der an dem gesamten Werke des Geistes auf die Tätigkeit des Individuums fällt, nur gering sein kann, so muss dieses, wie die Natur der Wissenschaft schon es mit sich bringt, sich umso mehr vergessen (Hegel 1807/1973, p. 66)”.

11 Fragment 50: “οὐκ ἐμοῦ, ἀλλὰ τοῦ λόγου ἀκούσαντας…” (‘By listening, not to me, but to reason…’). See for Instance Heidegger (1951/1954, p. 207).

12 The term κένωσις refers to a gesture of self-emptying or self-renunciation in order to achieve an ethos of servitude. Cf. Paul’s *Letter to the Philippians* (2:7).
